# SIRI as a Prognostic Marker in Metastatic Pancreatic Cancer

**DOI:** 10.3390/medicina61112020

**Published:** 2025-11-12

**Authors:** Hikmet Akar, Ferhat Ekinci, Atike Pınar Erdoğan, Mustafa Şahbazlar

**Affiliations:** Department of Medical Oncology, Faculty of Medicine, Manisa Celal Bayar University, Manisa 45030, Turkey; drferhatekinci@hotmail.com (F.E.); dr_pinarcan@yahoo.com (A.P.E.); m_sahbazlar@hotmail.com (M.Ş.)

**Keywords:** pancreatic cancer, systemic inflammation response index, SIRI, overall survival, prognostic marker

## Abstract

*Background and Objectives:* Systemic inflammation plays a critical role in cancer progression and prognosis. The Systemic Inflammation Response Index (SIRI), a novel marker integrating neutrophil, monocyte, and lymphocyte counts, has been suggested as a prognostic indicator in various malignancies. This study aimed to evaluate the prognostic significance of SIRI in patients with metastatic pancreatic cancer receiving first-line chemotherapy. *Materials and Methods:* This retrospective study included 147 patients with metastatic pancreatic cancer who received first-line chemotherapy or best supportive care between 2010 and 2024. Clinical and laboratory data were collected from medical records. Overall survival (OS) and progression-free survival (PFS) were assessed using the Kaplan–Meier method, and prognostic factors were identified by univariate and multivariate Cox regression analyses. *Results*: The median OS and PFS were 7 and 4 months, respectively. Multivariate analysis revealed that ECOG ≥ 2 (HR: 2.094, *p* = 0.019), liver metastasis (HR: 2.039, *p* = 0.027), and each unit increase in SIRI (HR: 1.156, *p* < 0.001) were independent predictors of poorer OS. Patients with SIRI > 1.86 had significantly shorter OS compared to those with SIRI ≤ 1.86 (median OS: 4 vs. 9 months, *p* = 0.019). *Conclusions*: SIRI is an independent prognostic marker for survival in metastatic pancreatic cancer patients undergoing first-line and subsequent lines of chemotherapy. These inflammation-based markers are simple, cost-effective tools that could be integrated into routine clinical practice to aid in risk assessment and treatment planning.

## 1. Introduction

Pancreatic cancer (PC), predominantly pancreatic ductal adenocarcinoma (PDAC), remains one of the most lethal malignancies worldwide, with a 5-year survival rate of approximately 10% [1]. According to the GLOBOCAN 2022 report, 510,992 new cases were diagnosed globally, ranking PC 12th among all cancers [2]. Recent global epidemiologic updates continue to show a rising incidence and mortality of pancreatic cancer despite advances in systemic therapies. The poor prognosis of this malignancy is primarily due to its early systemic dissemination, high metastatic potential, and resistance to conventional chemotherapy [3,4,5].

The aggressiveness and lethality of PDAC are largely determined by its highly desmoplastic and immunosuppressive tumor microenvironment, characterized by dense stroma, abundant inflammatory infiltrates, and immunosuppressive signaling that collectively promote tumor progression and therapeutic resistance [4]. This inflammatory milieu not only supports tumor growth but also impairs host antitumor immunity, emphasizing the central role of systemic inflammation in pancreatic cancer biology.

Systemic inflammation can be quantified using circulating cytokines, acute-phase proteins, and hematologic cell ratios, which have been shown to correlate with disease progression and survival in several malignancies [6]. Composite indices derived from complete blood counts, including the neutrophil-to-lymphocyte ratio (NLR), platelet-to-lymphocyte ratio (PLR), and prognostic nutritional index (PNI), reflect the balance between tumor-induced inflammation and host immune competence. Among these, the Systemic Inflammation Response Index (SIRI)—calculated as neutrophil × monocyte/lymphocyte—integrates both pro-tumor and antitumor immune elements, providing a broader representation of the inflammatory and immunologic state [7,8,9].

Previous studies have investigated the prognostic value of SIRI in resectable and locally advanced PC [10,11]. However, few have focused specifically on patients with metastatic pancreatic cancer, in whom systemic inflammation and immune dysfunction are most pronounced. Moreover, prior work primarily evaluated SIRI after chemotherapy, whereas its prognostic significance at the time of metastasis and before treatment initiation remains insufficiently characterized.

Although Qi et al. (2016) first described the prognostic value of SIRI in pancreatic cancer after chemotherapy [11], our study provides novel insights by evaluating SIRI at the time of metastasis in a real-world, contemporary cohort treated between 2010 and 2024. Unlike earlier studies, we focused exclusively on metastatic patients receiving first-line and subsequent lines of chemotherapy, analyzed detailed clinical and biochemical parameters, and determined the optimal SIRI cutoff using receiver operating characteristic (ROC) analysis. Therefore, this study expands existing evidence by confirming the independent prognostic value of SIRI in advanced-stage pancreatic cancer and by proposing its integration into routine clinical decision-making.

## 2. Materials and Methods

### 2.1. Clinical Characteristics of the Cohort

The retrospective study included 147 patients diagnosed with pancreatic adenocarcinoma who were followed up at the Medical Oncology Department of Manisa Celal Bayar University Hospital between 2010 and 2024. Diagnosis was validated histopathologically. Data were obtained from the hospital system and patients’ oncological records. All collected data were anonymized; therefore, informed consent was not required. This study was conducted in accordance with the Declaration of Helsinki.

The patients were staged using the TNM Classification of Malignant Tumours, 8th edition. In the diagnosis and follow-up of the patients, positron emission tomography, contrast-enhanced computed tomography, contrast-enhanced magnetic resonance imaging, and magnetic resonance cholangiopancreatography examinations were used as standard radiological examinations.

Data on age, gender, performance score, comorbidities, smoking and alcohol use status, symptoms at the time of diagnosis, tumor localization, stage at the time of diagnosis, and chemotherapy history were obtained from the patient files. Carcinoembryonic antigen (CEA), CA-19-9, albumin, globulin, C-reactive protein (CRP), total bilirubin, ALP, neutrophil, lymphocyte, and monocyte values were obtained from the laboratory records of the hospital automation system.

The cutoff value for age was determined as 65, for Eastern Cooperative Oncology Group (ECOG) performance score as 2, and for body mass index (BMI) classification as 25.

Patients with acute infections, recent surgical procedures (within 4 weeks), corticosteroid or granulocyte-colony stimulating factor (G-CSF) use, or any active inflammatory or autoimmune disorders were excluded. For patients presenting with biliary obstruction or cholangitis, baseline laboratory tests were obtained only after biliary drainage and normalization of infection markers (white blood cell count and C-reactive protein).

### 2.2. Biochemical Analysis and SIRI Calculation

SIRI was calculated using the following formula: SIRI = N × M/L, where N, M, and L represent peripheral neutrophil, monocyte, and lymphocyte counts at the time of metastasis, respectively. The optimal SIRI cutoff values for progression after chemotherapy in the cohort were determined using an ROC analysis. The results of the ROC analysis revealed that the optimal cutoff value for SIRI in this cohort was 1.86.

Progression-free survival (PFS) was calculated as the time interval between the initiation of treatment or best supportive care and the date of the first follow-up imaging showing disease progression. Progression was defined as an increase of more than 20% in the sum of the longest diameters of target lesions or the appearance of any new lesion. Overall survival (OS) was determined as the time from the date of diagnosis of metastasis to the date of death or, for patients who were still alive, the date of the last clinical follow-up.

Baseline hematologic parameters were obtained from blood samples drawn at the time of metastatic disease diagnosis, before initiation of any systemic chemotherapy or best supportive care.

### 2.3. Statistical Analysis

For statistical analysis, Statistical Package for the Social Sciences (SPSS) 15.0 (SPSS Inc., Chicago, IL, USA) was used. Descriptive statistics were presented as frequencies and percentages for categorical variables, and as mean, standard deviation, minimum, and maximum values for numerical variables. Survival rates were calculated using Kaplan–Meier analysis, while risk factors were assessed using Cox regression analysis. Because SIRI distribution was markedly right-skewed, it was log-transformed prior to inclusion in multivariate Cox regression models. This normalization reduced collinearity and resulted in a higher hazard ratio when adjusted for covariates. A statistical significance level of *p* < 0.05 was considered. Non-normally distributed variables, including SIRI and tumor markers, were presented as median (interquartile range). SIRI values were log-transformed for multivariate analyses to reduce skewness.

### 2.4. Statement on the Use of Artificial Intelligence

Artificial intelligence tools were used in a limited manner during the preparation of this manuscript. A large language model (ChatGPT, OpenAI, San Francisco, CA, USA; GPT-5) was employed to improve the linguistic clarity of the text, enhance coherence, and standardize English technical terminology. AI assistance was also utilized during the reference management process to check the accuracy of citations, match DOI numbers, and ensure consistency of reference formatting. No AI tools were used for data analysis, interpretation of results, or generation of scientific content. All AI-assisted outputs were carefully reviewed, verified, and edited by the authors, who take full responsibility for the integrity and accuracy of the final manuscript.

## 3. Results

In this study, the clinical and laboratory characteristics of 147 patients diagnosed with metastatic PC and treated with first-line chemotherapy at our center were retrospectively evaluated for their association with survival outcomes.

The median age at diagnosis was 63.2 years. Among the patients, 108 (73.5%) had an ECOG performance status of 0 or 1. A total of 57 patients (38.8%) had a BMI of 25 or greater. At baseline, 64 patients (43.5%) were active smokers, and 17 (11.6%) reported alcohol use. Regarding comorbidities, diabetes mellitus was present in 51 patients (34.7%) and coronary artery disease in 15 patients (10.2%). At diagnosis, abdominal pain was the most common symptom, observed in 138 patients (93.9%), followed by weight loss in 87 (59.2%) and jaundice in 32 (21.8%). Most patients (n = 117, 79.6%) presented with stage 4 disease. The primary tumor was located in the pancreatic head in 100 cases (68.0%). Surgical resection had not been performed in 102 patients (71.8%). The mean follow-up duration was 20.3 months. Review of outcomes showed that 135 patients (91.8%) had died during the follow-up period (Table 1). 11 patients had incomplete treatment-related records (missing data on chemotherapy administration or response) and were therefore excluded from treatment-specific analyses.

Baseline laboratory parameters are summarized in Table 2. The median (IQR) SIRI value was 1.94 (1.02–3.61), reflecting a right-skewed distribution consistent with chronic systemic inflammation. Mean CEA and CA 19-9 levels were elevated across the cohort, supporting the presence of high tumor burden and inflammatory activity.

**Figure 1:**
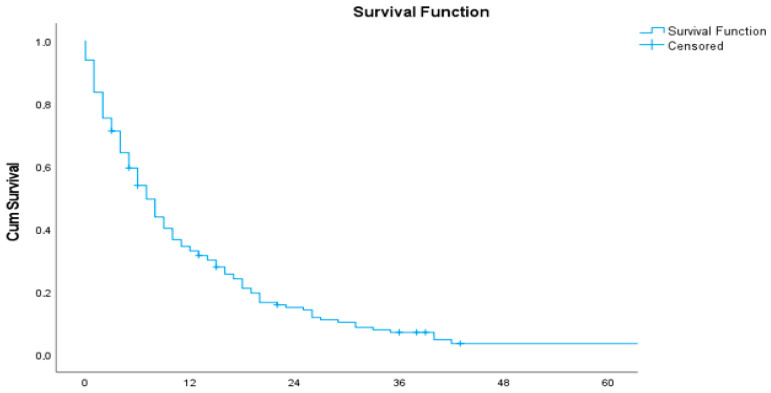
Overall survival (OS).

**Figure 2:**
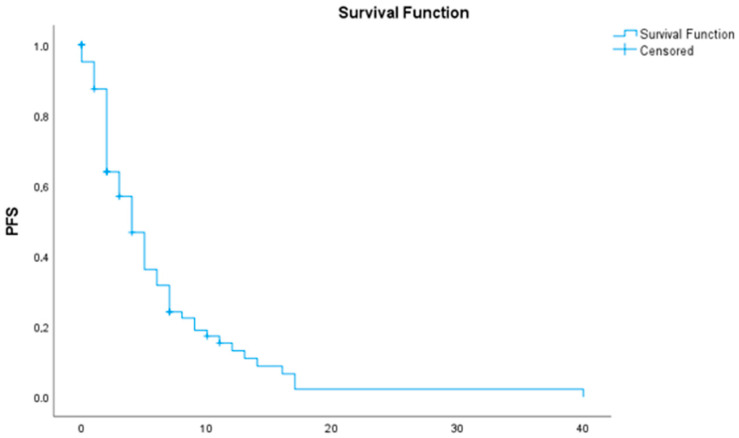
Progression free survival (PFS).

The median overall survival (OS) was 7 months (95% CI: 5.43–8.57) (Table 3 and Figure 1). The median progression-free survival (PFS) following first-line chemotherapy was 4 months (95% CI: 2.90–5.10) (Table 4 and Figure 2).

In the univariate Cox regression analysis, several clinical and laboratory factors were significantly associated with overall survival (OS). Patients with an ECOG performance status ≥ 2 exhibited a significantly increased risk of mortality compared to those with ECOG 0–1 (HR: 2.645, 95% CI: 1.782–3.925, *p* < 0.001). The presence of liver metastasis (HR: 2.113, 95% CI: 1.402–3.186, *p* < 0.001) and metastatic disease at diagnosis (HR: 2.414, 95% CI: 1.559–3.738, *p* < 0.001) were also associated with a poorer prognosis. Among inflammatory markers, each unit increase in SIRI was correlated with a statistically significant increase in mortality (HR: 1.001, 95% CI: 1.000–1.001, *p* < 0.001). Higher CEA levels (HR: 1.002, 95% CI: 1.000–1.003, *p* = 0.006), abdominal pain (HR: 2.525, 95% CI: 1.103–5.779, *p* = 0.028), and jaundice (HR: 1.685, 95% CI: 1.119–2.536, *p* = 0.012) were associated with worse OS. Conversely, patients who underwent prior surgery had a significantly lower risk of mortality (HR: 0.370, 95% CI: 0.243–0.565, *p* < 0.001). Nutritional and hepatic composite markers, such as the Albumin/ALP ratio (*p* = 0.005), were protective factors.

**Figure 3:**
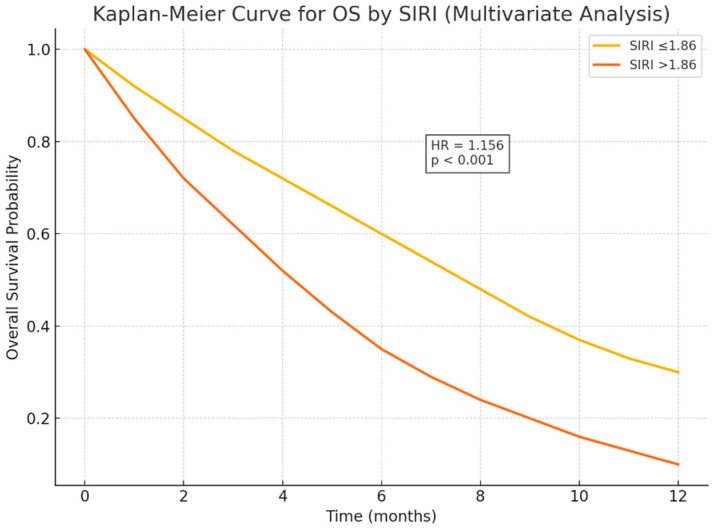
Kaplan–Meier curve by OS and SIRI.

In the multivariate analysis, only a subset of these factors remained statistically significant. Multivariate Cox regression analysis revealed that patients with ECOG performance status ≥ 2 had a 2.09-fold higher risk of mortality compared to those with ECOG 0–1 (*p* = 0.019). The presence of liver metastasis was associated with a 2.03-fold increased risk of death (*p* = 0.027). Additionally, each unit increase in the Systemic Inflammation Response Index (SIRI) was associated with a 1.156-fold increase in mortality risk (*p* < 0.001). Patients with a SIRI value > 1.86 had significantly shorter survival than those with SIRI ≤ 1.86 (median OS: 4 vs. 9 months, *p* = 0.019) (Table 5 and Figure 3).

In the univariate Cox regression analysis for progression-free survival (PFS), an increase in carcinoembryonic antigen (CEA) levels was significantly associated with a higher risk of disease progression (HR: 1.003, 95% CI: 1.001–1.004, *p* = 0.003), highlighting the adverse prognostic impact of tumor burden. Peritoneal metastasis showed a non-significant trend toward shorter PFS (HR: 1.559, 95% CI: 0.926–2.626, *p* = 0.095). Other clinical parameters, including ECOG performance status ≥ 2 (HR: 1.709, 95% CI: 0.872–3.350, *p* = 0.118) and jaundice (HR: 1.463, 95% CI: 0.791–2.707, *p* = 0.225), did not reach statistical significance in the univariate analysis.

**Figure 4:**
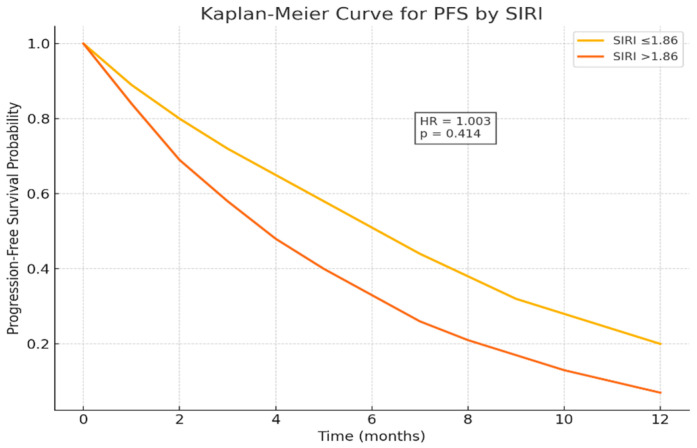
Kaplan–Meier curve for PFS by SIRI.

In the multivariate model, CEA remained an independent prognostic factor for PFS (HR: 1.003, 95% CI: 1.001–1.005, *p* = 0.007), confirming its role as a marker of tumor aggressiveness. ECOG ≥ 2 also retained borderline prognostic relevance, although not statistically significant (HR: 1.802, 95% CI: 0.851–3.816, *p* = 0.124). Peritoneal metastasis did not maintain independent significance in the multivariate analysis (*p* > 0.05), suggesting that their univariate effects may be influenced by other clinical variables (Table 6 and Figure 4).

When analyzed according to SIRI and the novel composite score reflecting tumor-related biological burden, patients with SIRI ≤ 1.86 had a median OS of 9 months and a 1-year OS rate of 38.5%. In contrast, those with SIRI > 1.86 had a median OS of only 4 months and a 1-year OS rate of 24.3%, suggesting a strong association between elevated inflammatory burden and poor prognosis. The 1-year OS rates were Kaplan–Meier estimates at 12 months with 95% confidence intervals (Table 7).

## 4. Discussion

This study demonstrates that the Systemic Inflammation Response Index (SIRI) serves as an independent prognostic biomarker in patients with metastatic pancreatic cancer receiving chemotherapy. Elevated SIRI levels (>1.86) were significantly associated with shorter overall survival (OS), confirming that systemic inflammation and immune suppression play pivotal roles in disease progression and treatment resistance. Although SIRI significantly predicted overall survival, it did not correlate with progression-free survival (*p* = 0.414), suggesting that it reflects long-term systemic inflammatory burden rather than short-term chemotherapy response.

The prognostic significance of SIRI aligns conceptually with other inflammation- and nutrition-based biomarkers, such as the Glasgow Prognostic Score (GPS), neutrophil-to-lymphocyte ratio (NLR), platelet-to-lymphocyte ratio (PLR), and prognostic nutritional index (PNI) [6,12,13]. GPS, which incorporates C-reactive protein (CRP) and albumin, has long been validated as a robust predictor of survival in gastrointestinal cancers. However, while GPS captures chronic inflammation and nutritional depletion, SIRI offers a more dynamic reflection of the immune–inflammatory equilibrium by integrating neutrophils, monocytes, and lymphocytes. Monocytes, in particular, contribute to tumor-associated macrophage formation and immunosuppression, thereby linking SIRI directly to tumor–immune interactions [14]. Thus, SIRI provides a more comprehensive view of host–tumor crosstalk and may complement traditional indices like GPS for refined prognostic stratification.

Interestingly, SIRI significantly predicted overall survival but not progression-free survival (*p* = 0.414). This finding suggests that SIRI primarily reflects the chronic systemic inflammatory status of the host rather than immediate chemotherapy responsiveness. Similar patterns have been reported in other gastrointestinal malignancies, where elevated inflammation indices were linked to post-progression rather than treatment-phase survival.

The mechanistic basis underlying the prognostic impact of SIRI lies in the dual role of inflammation and immunity within the PDAC microenvironment. Neutrophils and monocytes facilitate tumor proliferation, angiogenesis, and invasion by secreting cytokines, chemokines, and matrix-degrading enzymes, while lymphocytes mediate anti-tumor cytotoxicity [6,14]. Consequently, a high SIRI reflects both an enhanced pro-tumor inflammatory response and a diminished adaptive immune capacity. This imbalance is particularly relevant in pancreatic cancer, characterized by desmoplasia, hypoxia, and immune exclusion. The integration of SIRI with emerging immunologic biomarkers—such as PD-L1 expression, tumor-infiltrating lymphocytes, and cytokine profiles—could further elucidate the interplay between systemic inflammation and immune suppression in PDAC. The prognostic significance of SIRI for OS but not PFS may reflect its role as a marker of long-term systemic inflammation and immune exhaustion rather than short-term chemotherapy sensitivity. Chronic inflammatory status influences survival through sustained tumor-promoting cytokine release and immune suppression even after disease progression.

Pancreatic ductal adenocarcinoma develops within a dense desmoplastic stroma characterized by activated fibroblasts and tumor-associated macrophages that secrete pro-inflammatory cytokines such as interleukin-6 (IL-6) and tumor necrosis factor-α (TNF-α). These cytokines sustain systemic inflammation and immune suppression. Peripheral leukocyte-based indices such as SIRI serve as inexpensive surrogates for this cytokine-driven biology, providing a clinically accessible link between tumor microenvironment and host response.

From a clinical standpoint, SIRI represents an inexpensive, easily obtainable, and reproducible biomarker that can be derived from routine blood counts without additional cost. Its incorporation into baseline assessment and longitudinal monitoring could enhance risk stratification, early identification of aggressive disease, and prediction of treatment response. Patients with persistently elevated SIRI values may exhibit biologically aggressive tumors and might benefit from intensified therapeutic strategies or early inclusion in clinical trials targeting the inflammatory microenvironment.

Furthermore, our findings indicate that poor performance status (ECOG ≥ 2) and liver metastasis were also independent predictors of worse OS, consistent with established prognostic determinants in metastatic PC [4,5]. Elevated carcinoembryonic antigen (CEA) levels were additionally associated with shorter progression-free survival (PFS), reflecting higher tumor burden and more aggressive disease biology [15].

## 5. Strengths and Limitations

The strengths of this study include a relatively large single-institution cohort and the integration of both hematologic and biochemical parameters into multivariate survival analyses. However, its retrospective design and lack of external validation remain limitations. We acknowledge that the optimal SIRI cutoff (1.86) was derived and tested within the same dataset, which may introduce optimism bias. External validation in independent cohorts is warranted to confirm its generalizability. Another limitation is the missing treatment-related data in 11 patients, which slightly reduced the number of cases included in chemotherapy-specific analyses. However, this is unlikely to have affected the main findings since baseline characteristics were similar between patients with complete and incomplete treatment data. The retrospective design spanning 14 years led to unavoidable treatment heterogeneity. Differences in chemotherapy regimens (FOLFIRINOX vs. gemcitabine-based) could have influenced outcomes; however, SIRI retained prognostic significance regardless of treatment type. Although NLR, PLR, and GPS were mentioned, they were not calculated in this analysis; future studies should perform direct comparisons among these indices.

## 6. Future Directions

Prospective, multicenter studies are warranted to confirm the prognostic value of SIRI and explore its role in guiding treatment selection—particularly in the era of immunotherapy and precision oncology. Translational research combining SIRI dynamics with immune and genomic signatures may also help identify patient subsets most likely to benefit from novel anti-inflammatory or immune-modulatory interventions.

## 7. Conclusions

In summary, the Systemic Inflammation Response Index (SIRI) is a reliable, cost-effective, and clinically accessible prognostic biomarker in metastatic pancreatic cancer. Its integration with existing inflammation-based indices, such as GPS, could improve prognostic precision and aid in personalized treatment planning. Future translational and prospective studies should focus on validating its clinical utility and exploring its potential as a biomarker for treatment monitoring and immunotherapy responsiveness.

## Figures and Tables

**Table 1:** Demographic and clinical characteristics of the patients.

Characteristic		Value (n = 147)
Mean age at diagnosis (±SD, min–max)		63.2 ± 9.5 (38–88)
Age group, n (%)	<65 years	77 (52.4%)
	≥65 years	70 (47.6%)
ECOG performance status, n (%)	0–1	108 (73.5%)
	≥2	39 (26.5%)
BMI group, n (%)	<25	90 (61.2%)
	≥25	57 (38.8%)
Smoking status, n (%)	Current smoker	64 (43.5%)
	Never smoker	71 (48.3%)
	Former smoker	12 (8.2%)
Alcohol use, n (%)		17 (11.6%)
Presenting symptoms, n (%)	Jaundice	32 (21.8%)
	Abdominal pain	138 (93.9%)
	Weight loss	87 (59.2%)
	Ascites	2 (1.4%)
Comorbidities, n (%)	Diabetes mellitus	51 (34.7%)
	Coronary artery disease	15 (10.2%)
	Chronic lung disease	5 (3.4%)
Stage at diagnosis, n (%)	Stage 1-2-3	30 (20.4%)
	Stage 4	117 (79.6%)
Tumor location, n (%)	Head	100 (68.0%)
	Body	20 (13.6%)
	Tail	26 (17.7%)
	Head and body	1 (0.7%)
Surgery performed, n (%)	No	102 (71.8%)
	Yes	40 (28.2%)
Surgical margin status, n (%)	Negative	19 (47.5%)
	Positive	7 (17.5%)
	Not assessed	14 (35.0%)
Presence of metastasis at diagnosis, n (%)	No	37 (25.3%)
	Yes	109 (74.7%)
Sites of metastasis, n (%)	Liver	100 (69.9%)
	Lung	39 (27.3%)
	Brain	3 (2.1%)
	Bone	24 (16.8%)
	Peritoneum	48 (33.6%)
	Lymph nodes	115 (80.4%)
First-line chemotherapy received, n (%)	No	40 (29.5%)
	Yes	96 (70.5%)
Response to first-line chemotherapy, n (%)	PD	24 (25.0%)
	PR	62 (64.5%)
	SD	7 (7.2%)
	CR	3 (3.1%)
Lines of treatment, n (%)	0	40 (29.4%)
	1	54 (39.7%)
	≥2	42 (30.8%)
Follow-up time (months)		20.3 ± 7.0
Mortality, n (%)	Alive	12 (8.2%)
	Deceased	135 (91.8%)

Note: Data regarding chemotherapy line and response were unavailable for 11 patients due to incomplete medical records.

**Table 2:** Laboratory parameters of the patients at diagnosis.

Parameter	Value (Median [IQR])
CEA (ng/mL)	22.4 (5.8–73.6)
CA 19-9 (U/mL)	1540 (410–5880)
Albumin (g/dL)	3.6 (3.2–3.9)
Globulin (g/dL)	3.1 (2.7–3.5)
CRP (mg/L)	2.0 (0.8–4.6)
Total Bilirubin (mg/dL)	0.9 (0.5–2.1)
SIRI	1.94 (1.02–3.61)

**Table 3:** Median overall survival (OS).

Median Estimate	SE	95% CI	
		Min	Maks
7 months	0.80	5.43	8.57

**Table 4:** Progression free survival (PFS).

Median Estimate	SE	95% CI	
		Min	Maks
4 months	0.56	2.90	5.10

**Table 5:** Univariate and multivariate cox regression analysis for overall survival.

Variable	Univariate		Multivariate	
	HR (95% CI)	*p*-Value	HR (95% CI)	*p*-Value
Age ≥ 65 vs. <65	1.10 (0.78–1.55)	0.575	1.01 (0.98–1.03)	0.457
ECOG ≥ 2 vs. 0–1	2.64 (1.78–3.92)	<0.001	2.09 (1.12–3.88)	0.019
BMI ≥ 25 vs. <25	0.73 (0.51–1.04)	0.088	1.03 (0.61–1.74)	0.903
Jaundice (yes vs. no)	1.68 (1.11–2.53)	0.012	1.00 (0.51–1.99)	0.980
Abdominal pain (yes vs. no)	2.52 (1.10–5.77)	0.028	0.89 (0.24–3.36)	0.874
Metastasis at diagnosis (yes vs. no)	2.41 (1.55–3.73)	<0.001	0.89 (0.27–2.96)	0.857
CEA (per unit increase)	1.002 (1.000–1.003)	0.006	1.001 (0.999–1.002)	0.236
Liver metastasis (yes vs. no)	2.11 (1.40–3.18)	<0.001	2.03 (1.08–3.82)	0.027
Peritoneal metastasis (yes vs. no)	1.82 (1.27–2.62)	0.001	1.03 (0.59–1.82)	0.901
SIRI (log-transformed per unit increase)	1.001 (1.000–1.001)	<0.001	1.156 (1.072–1.247)	<0.001

**Table 6:** Multivariate Cox regression analysis for progression-free survival.

Variable	Univariate		Multivariate	
	HR (95% CI)	*p*-Value	HR (95% CI)	*p*-Value
Age ≥ 65 vs. <65	1.15 (0.70–1.88)	0.564	Not retained in final model (*p* > 0.05)	Not retained in final model (*p* > 0.05)
ECOG ≥ 2 vs. 0–1	1.70 (0.87–3.35)	0.118	1.80 (0.85–3.81)	0.124
BMI ≥ 25 vs. <25	1.25 (0.77–2.05)	0.361	Not retained in final model (*p* > 0.05)	Not retained in final model (*p* > 0.05)
Jaundice (yes vs. no)	1.46 (0.79–2.70)	0.225	1.56 (0.76–3.20)	0.220
Abdominal pain (yes vs. no)	1.11 (0.34–3.56)	0.858	0.89 (0.24–3.36)	0.874
Metastasis at diagnosis (yes vs. no)	1.38 (0.68–2.80)	0.368	Not retained in final model (*p* > 0.05)	Not retained in final model (*p* > 0.05)
CEA (per unit increase)	1.003 (1.001–1.004)	0.003	1.003 (1.001–1.005)	0.007
Liver metastasis (yes vs. no)	1.03 (0.59–1.80)	0.906	Not retained in final model (*p* > 0.05)	Not retained in final model (*p* > 0.05)
Peritoneal metastasis (yes vs. no)	1.55 (0.92–2.62)	0.095	1.48 (0.83–2.65)	0.181
SIRI (per unit increase)	1.000 (0.999–1.001)	0.414	1.003 (0.999–1.007)	0.414

**Table 7:** Survival according to SIRI.

Group	Median OS (Months)	1-Year OS Rate (%) [95% CI]
SIRI ≤ 1.86	9	38.5 [30.4–46.6]
SIRI > 1.86	4	24.3 [17.2–31.4]

## Data Availability

The data presented in this study are available upon request from the corresponding author. The data are not publicly available due to ethical and privacy restrictions.

## References

[1] Cai J., Chen H., Lu M., Zhang Y., Lu B., You L., Zhang T., Dai M., Zhao Y. (2021). Advances in the epidemiology of pancreatic cancer: Trends, risk factors, screening, and prognosis. Cancer Lett..

[2] Sung H., Ferlay J., Siegel R.L., Laversanne M., Soerjomataram I., Jemal A., Bray F. (2023). Global Cancer Statistics 2022: GLOBOCAN Estimates of Incidence and Mortality Worldwide for 36 Cancers in 185 Countries. CA Cancer J. Clin..

[3] Kolbeinsson H.M., Chandana S., Wright G.P., Chung M. (2022). Pancreatic Cancer: A Review of Current Treatment and Novel Therapies. J. Investig. Surg..

[4] Ettrich T.J., Seufferlein T. (2021). Systemic Therapy for Metastatic Pancreatic Cancer. Curr. Treat. Options Oncol..

[5] Wei L., Xie H., Yan P. (2020). Prognostic value of the systemic inflammation response index in human malignancy: A meta-analysis. Medicine.

[6] He Q., Li L., Ren Q. (2021). The prognostic value of preoperative Systemic Inflammatory Response Index (SIRI) in patients with high-grade glioma and the establishment of a nomogram. Front Oncol..

[7] Zhou Q., Su S., You W., Wang T., Ren T., Zhu L. (2021). Systemic Inflammation Response Index as a Prognostic Marker in Cancer Patients: A Systematic Review and Meta-Analysis of 38 Cohorts. Dose Response.

[8] Diakos C.I., Charles K.A., McMillan D.C., Clarke S.J. (2014). Cancer-related inflammation and treatment effectiveness. Lancet Oncol..

[9] Jia C.P., Chen H., Sun B. (2019). Research advances on the value of preoperative systemic inflammatory response index in predicting the prognosis of patients with resectable pancreatic cancer. Zhonghua Wai Ke Za Zhi..

[10] Wang D.S., Luo H.Y., Qiu M.Z., Wang Z.Q., Zhang D.S., Wang F.H., Li Y.H., Xu R.H. (2012). Comparison of the prognostic values of various inflammation based factors in patients with pancreatic cancer. Med. Oncol..

[11] Qi Q., Zhuang L., Shen Y., Geng Y., Yu S., Chen H., Liu L., Meng Z., Wang P., Chen Z. (2016). A novel systemic inflammation response index (SIRI) for predicting the survival of patients with pancreatic cancer after chemotherapy. Cancer.

[12] Onodera T., Goseki N., Kosaki G. (1984). Prognostic nutritional index in gastrointestinal surgery of malnourished cancer patients. Nihon Geka Gakkai Zasshi.

[13] McMillan D.C. (2013). The systemic inflammation-based Glasgow Prognostic Score: A decade of experience in patients with cancer. Cancer Treat. Rev..

[14] Kitsugi K., Kawata K., Noritake H., Chida T., Ohta K., Ito J., Takatori S., Yamashita M., Hanaoka T., Umemura M. (2024). Prognostic value of neutrophil to lymphocyte ratio in patients with advanced pancreatic ductal adenocarcinoma treated with systemic chemotherapy. Ann. Med..

[15] Kobayashi K., Ono Y., Kitano Y., Oba A., Sato T., Ito H., Mise Y., Shinozaki E., Inoue Y., Yamaguchi K. (2023). Prognostic Impact of Tumor Markers (CEA and CA19-9) on Patients with Resectable Colorectal Liver Metastases Stratified by Tumor Number and Size: Potentially Valuable Biologic Markers for Preoperative Treatment. Ann. Surg. Oncol..

